# Paternal separation as an independent risk factor for irritable bowel syndrome in rural Chinese left-behind children: a multicenter cross-sectional study

**DOI:** 10.3389/fpubh.2025.1592358

**Published:** 2025-07-21

**Authors:** Zhongcao Wei, Yu Zhang, Shaoxian Xu, Mei Tong, Xing Yang, Xin Xing, Fei Dai, Jinhai Wang, Bin Qin

**Affiliations:** ^1^Department of Gastroenterology, The Second Affiliated Hospital, Xi’an Jiaotong University, Xi'an, China; ^2^Shaanxi Key Laboratory of Gastrointestinal Motility Disorders, Xi'an, China; ^3^Shaanxi Provincial Clinical Research Center for Gastrointestinal Diseases, Xi'an, China; ^4^Digestive Disease Quality Control Center of Shaanxi Province, Xi'an, China; ^5^Department of Gastroenterology, Shaanxi Provincial People’s Hospital, The Third Affiliated Hospital, Xi’an Jiaotong University, Xi'an, China; ^6^School of Humanities and Social Sciences, Xi'an Jiaotong University, Xi'an, China; ^7^Department of General Medicine, The Second Affiliated Hospital, Xi’an Jiaotong University, Xi'an, China; ^8^Health Management Department, The Second Affiliated Hospital, Xi’an Jiaotong University, Xi'an, China

**Keywords:** irritable bowel syndrome, left-behind children, association, separation, risk factor

## Abstract

**Background:**

No study has assessed the relationship between separation factors and irritable bowel syndrome (IBS). We assess the association between the separation factors and IBS in Chinese left-behind children (LBC).

**Methods:**

In this school-based study, we analyzed data from eight representative primary and secondary schools to assess the association between separation factors and IBS in Chinese LBC based on the Rome IV criteria. While individual-level income data were unavailable, all analyses were adjusted for regional socioeconomic disparities (Guanzhong Basin vs. Northern Shaanxi vs. Southern Shaanxi).

**Results:**

A total of 349 IBS and 7,355 non-IBS children were included, and the proportion of LBC was 21.13%. Univariate analysis showed statistically significant differences in separation status between IBS and non-IBS children (*p* < 0.05). Multivariate analysis showed that in the model adjusted for demographic variables, separation from both parents demonstrated the strongest association with IBS (OR = 2.2, *p* < 0.0001), and separation from father only (OR = 2.1, *p* < 0.0001) was significantly positively associated with IBS, but separation from mother only was not significantly associated with IBS (*p* > 0.05), and the same trend was observed in the subgroup analysis of sex. The relationship between age and IBS risk in LBC was nonlinear, and the risk of IBS in LBC was highest at age 8 years.

**Conclusion:**

Separation from father only was significantly positively associated with IBS. And the relationship between age and IBS risk in LBC was nonlinear.

## 1 Introduction

Due to rapid urbanization in China, large numbers of young people migrate to large cities from rural areas to find better jobs. They invariably leave their children in the care of another parent or the grandparents, leading to a unique phenomenon called left behind child (LBC) (1). LBCs were defined as children under 18 years old who were separated from one or both of their parents for more than 6 months (2). It was estimated that more than sixty million LBCs exist in rural China. A large number of studies have demonstrated that the health of LBCs was affected; specifically, an increased risk of nutritional, developmental and mental health problems was found to be related to parental migration in LBCs (3–5). However, little attention has been given to the disorders of gut brain interaction related to parental separation (4).

Irritable bowel syndrome (IBS), characterized by abdominal pain and altered bowel habits, is one of the most common functional astrointestinal diseases in children and adolescents. The prevalence of IBS in children has been reported to be as high as 2.8–25.7% (6, 7), which influences the quality of school life in adolescents and sometimes requires medical intervention (8). It is regarded as a stress-sensitive disorder of brain-gut interactions with an increased prevalence of childhood adverse events, such as parental separation (9, 10); other factors include intestinal infection, socioeconomic conditions, food habits, sex and other factors (11, 12). Socioeconomic factors, including family income and academic performance, have been suggested as potential risk factors for IBS (13). Specifically, Madhusudan et al. (14) demonstrated that low family income was significantly associated with higher odds of IBS (OR = 2.4; 95% CI:1.2–4.9). LBC, a vulnerable population in China, face additional socioeconomic disadvantages, including poorer health behaviors, lower school engagement, and a higher prevalence of depression compared to their non-LBC counterparts (2). Notably, depression rates among LBC show an inverse correlation with household income levels (2). However, the association between socioeconomic status and IBS remains understudied in the context of Chinese LBC, despite their unique psychosocial stressors and economic vulnerability.

Maternal separation in rodents is an excellent model of early life stress for the study of brain-gut interaction disorders, such as IBS, which is characterized by alterations in intestinal barrier function, an imbalance in enteric microflora and visceral hypersensitivity (15). Thus, we speculated that parental separation might influence gastrointestinal function in LBCs; however, the relationship between separation factors and IBS has not yet been assessed in Chinese children.

The disorders of gut brain interaction in adolescents are mostly diagnosed based on the Rome criteria, and in 2016 (16), the Rome IV criteria were published. And few studies have established the prevalence of IBS in children and adolescents using the Rome IV criteria. In the present study, we assessed the incidence of IBS in Chinese LBC based on the Rome IV criteria and sought for the first time to analyze the association between the separation factors and IBS.

## 2 Methods

### 2.1 Participants

A cross-sectional study was conducted between March 2021 and September 2021 in Shaanxi Province in China. We chose the northern Shaanxi, southern Shaanxi and Guanzhong basin regions of Shaanxi Province, which are representative of the different geographic and dietary backgrounds of China. The sampling employed a two-stage stratified random design: first by socioeconomic region (Northern Shaanxi, Southern Shaanxi, and Guanzhong Plain), then by school level within each region. Using R software for randomization, we selected a total of 9 schools comprising 4 senior schools (grades 10–12, ages 15–17), 2 junior schools (grades 7–9, ages 12–14) and 3 primary schools (grades 1–6, ages 6–11). The students were invited to complete a self-report questionnaire based on the pediatric Rome IV criteria, which was translated to a Chinese version (17, 18). The students had no time limitation to fill in the questionnaires under the guidance of trained research personnel. All research personnel received training in advance. When students completing the questionnaire, if necessary, a research personnel will be present to provide assistance. If there were questions in the questionnaire that children cannot determine, they should be clarified by consulting parents or teachers. Written informed consent from the children and their parents was obtained before the survey. The study protocol conforms to the ethical guidelines of the 1975 Declaration of Helsinki (6th revision, 2008), and ethical approval was obtained from the ethics committee of the Second Affiliated Hospital of Xi’an Jiaotong University.

### 2.2 Inclusion and exclusion criteria

The inclusion criteria were children (ages 6 to 17) from enrolled in school. Children were excluded if they were aged ≥18 years, had known neurological or psychiatric disorders, recently took anti-anxiety drugs or anti-depressant drugs, had a history of abdominal surgery, had a peptic ulcer or ulcerative colitis, had metabolic diseases, such as diabetes mellitus or hyperthyroidism, did not complete the questionnaire, did not accept participation in the study or had incomplete data.

### 2.3 Data collection

Demographic data, such as age, sex, height (in meters), weight (in kilograms), school type and region, were collected using the questionnaire. Data about a history of breastfeeding, long school accommodation (staying at school more than 5 days per week) and prolonged school meals (eating at school more than 5 days per week) as well as indicators related to parental separation were also collected to analyze the relationship between separation factors and IBS (19). Pediatric IBS was classified into 4 subgroups according to the predominant stool patterns: diarrhea-predominant IBS subgroup (IBS-D), constipation-predominant IBS subgroup (IBS-C), mixed bowel habits IBS subgroup (IBS-M) and unclassified IBS subgroup (IBS-U). All questionnaire data were imported into the database by a trained researcher.

### 2.4 Sample size

The sample size was calculated based on the prevalence of IBS in children, 2.8–25.7% (6, 7). Specifically, we selected the lower incidence of 5% as the calculation criterion, with a 1% margin of error and 95% confidence level. Using the PASS 15.0 software, we calculated that at least 7,501 children needed to be screened for this study (20). Assuming that the invalid questionnaire rate was 20%, the sample size increased to 9,001. In the study, we eventually surveyed 9,307 children in China.

### 2.5 Definition of IBS

Rome IV IBS was defined as recurrent abdominal pain for at least 4 days per month in the last 2 months associated with 1 or more of the following: improvement in abdominal pain or discomfort after defecation, onset associated with a change in frequency of stool, onset associated with a change in form (appearance) of stool, and symptoms cannot be fully explained by other diseases (16).

### 2.6 Statistical analysis

The EpiData3.1 software was used to input the data, and the PASS15 software was used to calculate the sample size. EmpowerStats and SPSS 22.0 were used for the statistical analysis. All categorical variables are reported as counts with percentages, and statistical testing was performed with the chi-square test or Fisher’s exact test, phi or Cramer V values were used to represent the effect size. For all continuous variables, means ± standard deviations (SD) were reported, statistical testing was performed with a t test or Kruskal–Wallis test, and Cohen’s d values were used to represent the effect size. And Effect sizes are indicated as small (0.2 ≤ effect size < 0.5), medium (0.5 ≤ effect size < 0.8), and large (effect size ≥ 0.8) (21). Multivariate analysis was used to assess the association between separation factors and IBS. Subgroup and interaction analyses were conducted according to sex (male and female) (22, 23). Smooth curve fitting was used to address the nonlinear association between age and IBS on the basis of LBC, adjusting for sex, BMI, region, history of breastfeeding, long school accommodation, and prolonged school meals. *p* < 0.05 was considered statistically significant.

## 3 Results

### 3.1 Baseline characteristics

**Figure 1:**
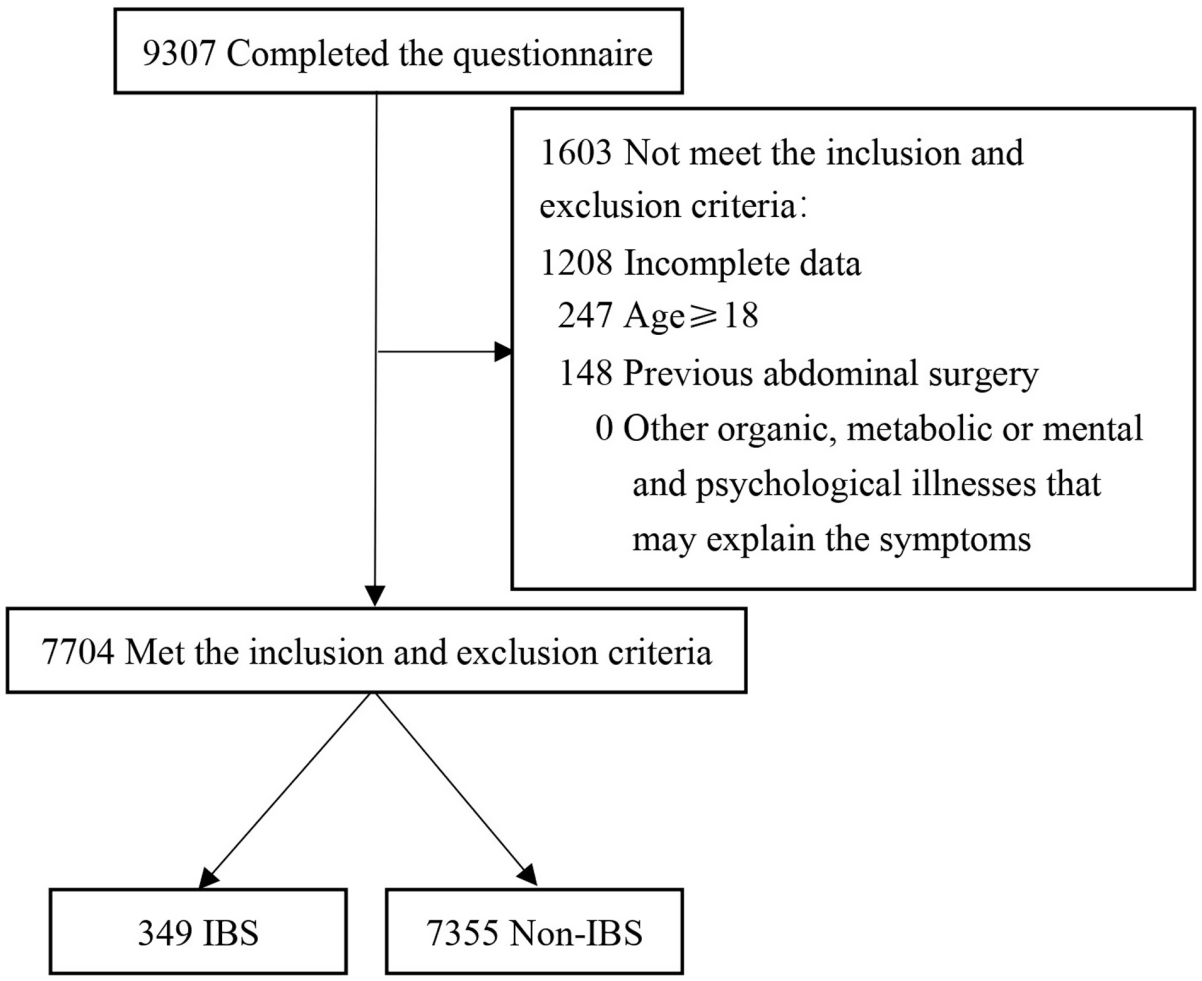
Flow chart of the study. IBS, irritable bowel syndrome.

**Figure 2:**
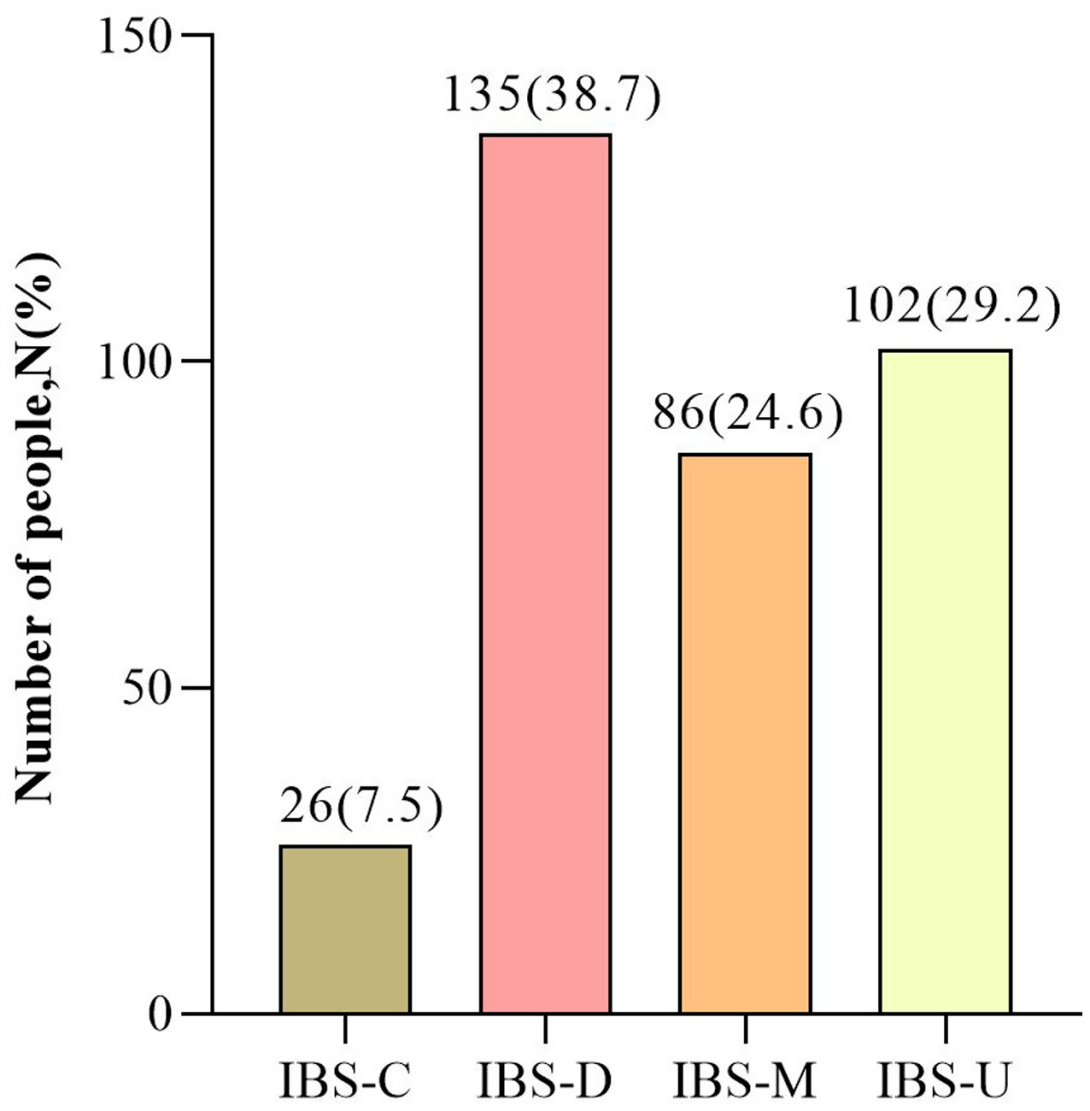
The Rome IV IBS subtype. IBS, irritable bowel syndrome; IBS-C, irritable bowel syndrome with constipation; IBS-D, irritable bowel syndrome with diarrhea; IBS-M, irritable bowel syndrome with mixed stool; IBS-U, undifferentiated irritable bowel syndrome.

Between March 2021 and September 2021, a total of 9,307 students completed the questionnaire. After excluding 1,603 students who did not meet the inclusion criteria or met the exclusion criteria, 7,704 children were eligible for inclusion in the study, including 349 IBS (4.53%) and 7,355 non-IBS (95.47%) children. The screening flow chart is shown in Figure 1. The mean age of the children included in the study was 13.97 ± 2.71 years, and the ages ranged from 6 to 17 years. Of these children, 1,563 were in primary school, 1813 were in junior high school and 4,328 were in senior high school. Among the included children, 1,628 were LBCs and 6,076 were non-LBCs, corresponding to a proportion of LBC of 21.13%. The LBCs were mainly separated from both parents; there were 2,113 children, accounting for 27.43% of the enrolled children. Regarding the Rome IV IBS subtype, 26 cases of IBS with constipation (IBS-C), 135 cases of IBS with diarrhea (IBS-D), 86 cases of IBS with mixed stool (IBS-M) and 102 cases of undifferentiated IBS (IBS-U) were included in the cohort (Figure 2). The baseline characteristics of the included children are summarized in Table 1.

**Table 1:** Baseline characteristics and univariate analysis of IBS and non-IBS.

Characteristics	All participants	Non-IBS	IBS	*p* value
	(*n* = 7,704)	(*n* = 7,355)	(*n* = 349)	
Age (y)	14.0 ± 2.7	13.9 ± 2.7	14.8 ± 2.6	<0.001
BMI (kg/m2)	20.0 ± 4.1	19.9 ± 4.1	20.0 ± 4.0	0.245
Sex				0.068
Male	3,878 (50.3)	3,719 (50.6)	159 (45.6)	
Female	3,826 (49.7)	3,636 (49.4)	190 (54.4)	
School type				<0.001
Primary school	1,563 (20.3)	1,511 (20.5)	52 (14.9)	
Middle school	1813 (23.5)	1771 (24.1)	42 (12.0)	
High school	4,328 (56.2)	4,073 (55.4)	255 (73.1)	
Region				<0.001
Northern Shaanxi	3,187 (41.4)	3,010 (40.9)	177 (50.7)	
Southern Shaanxi	1,452 (18.8)	1,424 (19.4)	28 (8.0)	
Guanzhong Basin	3,065 (39.8)	2,921 (39.7)	144 (41.3)	
History of breastfeeding				0.327
No	1,077 (14.0)	1,022 (13.9)	55 (15.8)	
Yes	6,627 (86.0)	6,333 (86.1)	294 (84.2)	
Long school accommodation				<0.001
No	4,221 (54.8)	4,113 (55.9)	108 (30.9)	
Yes	3,483 (45.2)	3,242 (44.1)	241 (69.1)	
Prolonged school meals				<0.001
No	3,365 (43.7)	3,288 (44.7)	77 (22.1)	
Yes	4,339 (56.3)	4,067 (55.3)	272 (77.9)	
Separation from parents				<0.001
No	4,696 (61.0)	4,567 (62.1)	129 (37.0)	
Yes	3,008 (39.0)	2,788 (37.9)	220 (63.0)	
Separation time				<0.001
0	4,696 (61.0)	4,567 (62.1)	129 (37.0)	
1–3 month	710 (9.2)	676 (9.2)	34 (9.7)	
3–6 month	670 (8.7)	622 (8.5)	48 (13.8)	
6–9 month	462 (6.0)	432 (5.9)	30 (8.6)	
>9 month	1,166 (15.1)	1,058 (14.4)	108 (30.9)	
Causes of separation				<0.001
No separation	4,696 (61.0)	4,567 (62.1)	129 (37.0)	
Live in school	1,066 (13.8)	981 (13.3)	85 (24.4)	
Parents’ migrant work	1724 (22.4)	1,617 (22.0)	107 (30.7)	
Parental divorce	184 (2.4)	165 (2.2)	19 (5.4)	
Parental death	34 (0.4)	25 (0.3)	9 (2.6)	
Separation status				<0.001
No separation	4,696 (61.0)	4,567 (62.1)	129 (37.0)	
Father only	700 (9.1)	657 (8.9)	43 (12.3)	
Mother only	195 (2.5)	185 (2.5)	10 (2.9)	
Both	2,113 (27.4)	1946 (26.5)	167 (47.9)	
LBC				<0.001
No	6,076 (78.9)	5,865 (79.7)	211 (60.5)	
Yes	1,628 (21.1)	1,490 (20.3)	138 (39.5)	

Values are expressed as the mean ± SD or n (%). LBC: left-behind children; BMI: body mass index; OR = odds ratio; CI = confidence interval; IBS: Irritable bowel syndrome.

### 3.2 Univariate analysis of IBS and non-IBS

The study included 349 Rome IV IBS paticipants and 7,335 non-IBS paticipants. A univariate analysis revealed statistically significant differences in age, school type, region, long school accommodation, and prolonged school meals between Rome IV IBS and non-IBS children (*p* < 0.001). Compared with non-IBS children, IBS children had a higher rate of separation from their parents (χ^2^ = 88.418, effect size = 0.107, *p* < 0.001), and compared with non-LBCs, LBCs had a higher incidence of IBS (χ^2^ = 74.338, effect size = 0.098, *p* < 0.001). Separation from fathers only, mothers only, and both parents were more common in IBS children than in non-IBS children, and the difference was statistically significant (*p* < 0.001). In addition, the length of separation (χ^2^ = 109.876, effect size = 0.119, *p* < 0.001) and reasons for separation (χ^2^ = 127.090, effect size = 0.128, *p* < 0.001) significantly differed between IBS children and non-IBS children. However, these statistically significant differences can all be considered small differences in the assessment of clinical relevance by effect sizes. The univariate analysis of IBS and non-IBS is shown in Table 1.

### 3.3 Multivariate analysis of IBS and non-IBS

The univariate analysis showed that separation factors significantly differed between children with IBS and non-IBS children, and we used multivariate analysis to further evaluate the association between separation factors and IBS. The multivariate analysis results of IBS and non-IBS children are shown in Table 2. Multivariate analysis showed that separation from parents and LBC were significantly positively associated with IBS in the unadjusted model, the model adjusted for age/sex, and the model adjusted for age, sex, BMI, school type, region, history of breastfeeding, long school accommodation, and prolonged school meals (*p* < 0.0001).

**Table 2:** Multivariate analysis of separation factors and IBS.

Separation factors	Model 1		Model 2		Model 3	
	OR (95% CI)	*P* value	OR (95% CI)	*P* value	OR (95% CI)	*P* value
Separation from parents						
No	1.0		1.0		1.0	
Yes	2.8 (2.2, 3.4)	<0.0001	2.5 (2.0, 3.1)	<0.0001	2.2 (1.8, 2.8)	<0.0001
Separation time						
0	1.0		1.0		1.0	
1–3 month	1.8 (1.2, 2.6)	0.0034	1.6 (1.1, 2.3)	0.0272	1.5 (1.0, 2.2)	0.0591
3–6 month	2.7 (1.9, 3.8)	<0.0001	2.4 (1.7, 3.4)	<0.0001	2.1 (1.5, 3.0)	<0.0001
6–9 month	2.5 (1.6, 3.7)	<0.0001	2.3 (1.5, 3.4)	<0.0001	2.0 (1.3, 3.0)	0.0012
>9 month	3.6 (2.8, 4.7)	<0.0001	3.3 (2.5, 4.3)	<0.0001	2.8 (2.1, 3.7)	<0.0001
Causes of separation						
No separation	1.0		1.0		1.0	
Live in school	3.1 (2.3, 4.1)	<0.0001	2.3 (1.7, 3.1)	<0.0001	1.9 (1.4, 2.6)	<0.0001
Parents’ migrant work	2.3 (1.8, 3.0)	<0.0001	2.3 (1.8, 3.0)	<0.0001	2.1 (1.6, 2.8)	<0.0001
Parental divorce	4.1 (2.5, 6.8)	<0.0001	4.0 (2.4, 6.7)	<0.0001	3.5 (2.1, 5.9)	<0.0001
Parental death	12.7 (5.8, 27.9)	<0.0001	13.8 (6.2, 30.6)	<0.0001	10.4 (4.6, 23.3)	<0.0001
Separation status						
No separation	1.0		1.0		1.0	
Father only	2.3 (1.6, 3.3)	<0.0001	2.3 (1.6, 3.2)	<0.0001	2.1 (1.5, 3.0)	<0.0001
Mother only	1.9 (1.0, 3.7)	0.0539	2.0 (1.0, 3.9)	0.0428	1.8 (0.9, 3.6)	0.0754
Both	3.0 (2.4, 3.8)	<0.0001	2.6 (2.1, 3.4)	<0.0001	2.2 (1.8, 2.9)	<0.0001
LBC						
No	1.0		1.0		1.0	
Yes	2.6 (2.1, 3.2)	<0.0001	2.4 (1.9, 3.0)	<0.0001	2.1 (1.7, 2.6)	<0.0001

Model 1: unadjusted; Model 2: adjusted for age and sex; Model 3: adjusted for age, sex, BMI, school type, region, history of breastfeeding, long school accommodation, and prolonged school meals.

The association analysis between separation time and IBS found that separation times of 3–6 months, 6–9 months and > 9 months were significantly positively associated with IBS in the unadjusted model, the model adjusted for age/sex and the model adjusted for demographic variables. Specifically, the association between separation time > 9 months and IBS was the highest, and this difference was statistically significant (*p* < 0.05).

An analysis of the association between causes of separation and IBS showed that living in school, parents’ migrant work, parental divorce, and parental death were all significantly positively associated with IBS in the unadjusted model, the model adjusted for age/sex and the model adjusted for demographic variables (*p* < 0.05). Among these causes of separation, the association between parental death and IBS was much higher than others, with an OR value of 10.4 (95% CI: 4.6–23.3, *p* < 0.0001) in the model adjusted for demographic variables. Given the unclear treatment approach for cases involving parental death, we performed a separate analysis of these cases (Supplementary Tables 1, 2). The results were consistent with those from the main analysis.

The analysis of separation status and IBS showed that separation from the father only and separation from both parents were significantly positively associated with IBS in the unadjusted model, the model adjusted for age/sex, and the model adjusted for demographic variables (*p* < 0.05). However, in the model adjusted for demographic variables, separation from mother only and IBS were not significantly associated (OR = 1.8, 95% CI: 0.9–3.6, *p* = 0.0754). Among these separation states, separation from both parents was more associated with IBS than separation from the father only, with an OR value of 2.2 (95% CI: 1.8–2.9, *p* < 0.0001).

### 3.4 Sex subgroup analysis

To assess the relationship between separation factors and IBS in different sexes, we performed subgroup and interaction analyses for sex. This subgroup analysis showed that separation from parents, LBC, separation time, cause of separation, and separation status were significantly positively associated with IBS in both male and female children after adjusting for age, BMI, school type, region, history of breastfeeding, long school accommodation, and prolonged school meals (*p* < 0.05). Notably, parental divorce significantly positively associated with IBS in female children compared with no separation (OR = 4.8, 95% CI: 2.5–9.0, *p* < 0.0001), while in male children, parental divorce and IBS were not associated (OR = 2.0, 95% CI: 0.8–5.2, *p* = 0.1489). Separation from fathers only was positively associated with IBS in both male (OR = 1.9, 95% CI: 1.1–3.2, *p* = 0.0219) and female (OR = 2.4, 95% CI: 1.5–3.9, *p* = 0.0005) children, while separation from mothers only was not significantly associated with IBS in either male or female children (*p* > 0.05). The sex subgroup analysis of the relationship between separation factors and IBS is shown in Table 3.

**Table 3:** Sex subgroup analysis of the relationship between separation factors and IBS.

Separation factors	Male		Female	
	OR (95% CI)	*P* value	OR (95% CI)	*P* value
Separation from parents				
No	1.0		1.0	
Yes	2.2 (1.5, 3.1)	<0.0001	2.2 (1.6, 3.0)	<0.0001
Separation time				
0	1.0		1.0	
1–3 month	1.3 (0.7, 2.4)	0.3467	1.6 (0.9, 2.7)	0.0928
3–6 month	2.0 (1.2, 3.4)	0.0118	2.2 (1.4, 3.5)	0.0008
6–9 month	2.0 (1.1, 3.7)	0.0243	2.0 (1.1, 3.5)	0.0205
>9 month	3.0 (2.0, 4.5)	<0.0001	2.7 (1.8, 4.0)	<0.0001
Causes of separation				
No separation	1.0		1.0	
Live in school	2.0 (1.3, 3.2)	0.0031	1.9 (1.3, 2.9)	0.0024
Parents’ migrant work	2.2 (1.5, 3.2)	<0.0001	2.0 (1.4, 3.0)	0.0003
Parental divorce	2.0 (0.8, 5.2)	0.1489	4.8 (2.5, 9.0)	<0.0001
Parental death	11.0 (3.3, 37.0)	0.0001	11.0 (3.7, 32.9)	<0.0001
Separation status				
No separation	1.0		1.0	
Father only	1.9 (1.1, 3.2)	0.0219	2.4 (1.5, 3.9)	0.0005
Mother only	1.6 (0.6, 4.0)	0.3630	2.1 (0.8, 5.3)	0.1370
Both	2.4 (1.6, 3.4)	<0.0001	2.2 (1.6, 3.0)	<0.0001
LBC				
No	1.0		1.0	
Yes	2.3 (1.6, 3.2)	<0.0001	2.0 (1.4, 2.7)	<0.0001

The multivariable model was adjusted for age, BMI, school type, region, history of breastfeeding, long school accommodation, and prolonged school meals.

### 3.5 The relationship between age and IBS based on LBC

**Figure 3:**
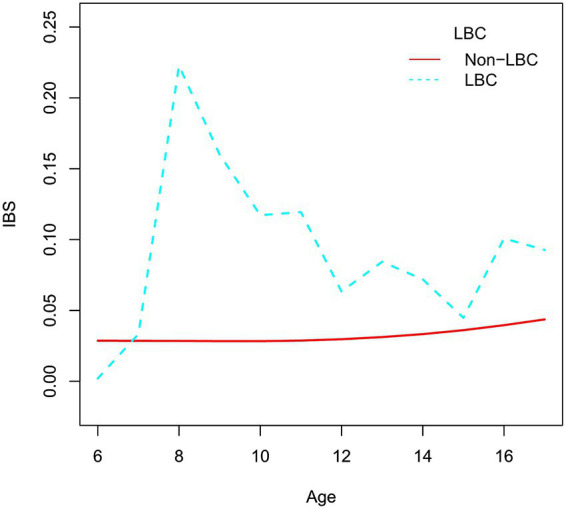
The relationship between age and IBS on the basis of LBC. IBS, irritable bowel syndrome; LBC, left behind child.

Multivariate analysis showed that LBC was significantly positively associated with IBS in the model adjusted for demographic variables, and we further assessed the relationship between age and IBS on the basis of LBC using smooth curve fitting (Figure 3). We found that the relationship between age and IBS risk in LBC was nonlinear (adjusted for sex, BMI, region, history of breastfeeding, long school accommodation, and prolonged school meals). At age 8 years, the risk of IBS in LBC was highest, whereas the risk of IBS in LBCs tends to decrease with age thereafter.

## 4 Discussion

This study appears to be the first to analyze the association between the separation factors and IBS in Chinese LBC using a school-based study. Our study showed that separation from parents, LBC, separation time, cause of separation were significantly positively associated with IBS. And separation from father only was significantly positively associated with IBS, while no association was found between separation from mother only and IBS. Our research highlights the impact of separation factors on IBS, especially the impact of separation from father only.

Due to the high proportion of LBCs in China, separation from parents can be a high-stress event that requires children to adjust and can lead to serious mental, psychological and behavioral problems. While prior studies of Chinese children have focused on nutrition and psychology, few studies have focused on gastrointestinal diseases and symptoms (2, 3, 24). In this multicenter cross-sectional trial, 7,704 children were eligible for inclusion in the study, and the incidence of LBC was 21.13%. We showed that compared with non-LBCs, LBC presented more frequently with Rome IV IBS, and LBC was significantly positively associated with IBS.

According to the 2010 national surveys of China, LBCs accounted for 21.88% of China’s children (25), and this LBC proportion was similar to that observed in our study. In our study, the incidence of Rome IV IBS was 4.58%. In a meta-analysis of 16 cross-sectional studies to evaluate the prevalence of IBS, the prevalence of IBS in Asian children varied from 2.8 to 25.7% (6). The IBS proportion in our study was remarkably comparable to that in other studies.

Separation from parents, LBC, separation time, cause of separation were significantly positively associated with IBS based on the Rome IV criteria in this study. Previous studies showed that compared with non-LBC, the brain structure of LBCs was altered and may result in mental disorders and psychosocial problems that lead to negative life outcomes (4, 5). Psychological disturbances are related to the pathophysiology of IBS (26). Since disturbances in brain-gut function are one of the physiological mechanisms of IBS (27), separation factors may be related to the occurrence of IBS. The results of the subgroup analysis by sex showed a significant positive association between parental divorce and IBS in female children but not in male children. This difference may arise because female children are more susceptible to emotional problems than male children (1) and are more susceptible to parental divorce.

Separation from parents may lead to interference in family routines, a lack of parental supervision, a weak relationship with parents, and reduced financial resources, which may lead to mental and psychological problems in children (28). Compared with separation from the mother only, separation from the father only appeared to have a greater association with IBS, possibly because of differences in caregiving roles for fathers and mothers (29). Parent–child play was related to children’s social skills, emotional skills and self-regulation. Father-child paly can promote children’s development and was associated with positive results over time (30). And compared with mothers, fathers challenged and let children take the lead in games. This sense of agency in the game might support children to use their developing vocabulary (30). Natasha et al. showed that the quality of father-child play was related to children’s stronger social emotional ability and emotional regulation, and could reduce children’s aggression, anxiety and negative emotions (31). Therefore, in terms of parent–child play, fathers play a larger role, and accordingly, separation from fathers seems to have a greater impact on children.

Previous studies have shown that children living in poverty may benefit more from the active involvement of their fathers than from their mothers, and migrant fathers provide less warmth and emotional support to their children than migrant mothers (29, 32). This may be another reason why separation from fathers alone had a greater impact on children. In our study, separation from mother only was positively associated with IBS, but there was no statistically significant difference, possibly due to the small number of children separated from mother only, accounting for 2.5% (195/7704). In the future, with the further increase of sample size, there may be a statistical difference. Our study highlighted the association between separation from father only and IBS, compared to separation from mother only.

In our study, the relationship between age and IBS risk in LBC was nonlinear. At age 8 years, the risk of IBS in LBC was highest, and this risk tended to decrease with age thereafter. This trend may have arisen because the development of children’s cognitive function is a continuous process (33). At the age of 6 or 7 years, cognitive function remains very poor, so separation from one’s father or mother had little effect on children. When the age reached 8 years old, children’s cognitive function gradually improved, but because they were very young and needed more care, separation from their father or mother had a greater impact on children. As the children aged, they gained independence to take care of themselves, and the degree of influence gradually decreased. While no prior studies have specifically assessed the age-IBS relationship in LBC, emerging evidence suggests that IBS—a disorder of the gut-brain axis with potential autonomic nervous system involvement—exhibits distinct age-dependent trends in pediatric populations. For instance, Paola et al. reported heightened autonomic symptom burden in children with IBS, supporting its neurophysiological underpinnings (34). Notably, retrospective data show that functional gastrointestinal disorders (FGIDs) peak in prevalence among 4–10-year-olds compared to younger (0–3 years) or older (11–15 years) children (35). This aligns with an epidemiological survey of Suzhou primary school students (grades 1–6), where IBS prevalence peaked at age 8 and declined gradually through ages 8–13—a trend consistent with our findings (36).

Our study had several limitations. First, our study was a cross-sectional study, which cannot evaluate the causal relationship between separation factors and IBS. Second, questionnaires were used to collect data in this study, which may have led to information bias. Third, this study was only conducted in Shaanxi Province, which might limit the universality of the results in the entire country. Future research should be conducted in a wider area to strengthen the existing research results. Fourth, although the number of parental separation factors in our study was comparable to other studies (5, 24, 37), there may still be some separation factors that have not been taken into account, and our main purpose was to evaluate the relationship between separation factors and IBS, no factors related to emotional disorders were included in the study, and the relationship between emotional disorders and IBS in LBC could not be evaluated, which is still a research direction worthy of further exploration in the future. Fifth, while regional stratification provides a proxy for socioeconomic differences, the absence of direct household-level economic data may affect the precision of our estimates. Future studies would benefit from incorporating more granular socioeconomic measures at the individual level.

In conclusion, this study was the first to explore the relationship between separation factors and IBS, and the results showed that separation from father only was significantly positively associated with IBS, while no association was found between separation from mother only and IBS. And parental divorce was not significantly associated with IBS in male children. The relationship between age and IBS risk in LBC was nonlinear.

## Data Availability

The raw data supporting the conclusions of this article will be made available by the authors, without undue reservation.
