# Supplementary material for: Paternal separation as an independent risk factor for irritable bowel syndrome in rural Chinese left-behind children: a multicenter cross-sectional study
Source: Front Public Health. 2025 Jul 21;13:1592358. doi: 10.3389/fpubh.2025.1592358 (PMC12318998; doi:10.3389/fpubh.2025.1592358)
Supplement: Supplementary file 1 [file Table_1.DOCX]

Supplementary Material

# Supplementary Tables

Supplementary Tables 1. Baseline characteristics and univariate analysis of IBS and non-IBS.

| Characteristics | All participants | Non-IBS | IBS | P value |
| --- | --- | --- | --- | --- |
|  | (n=7704) | (n=7355) | (n=349) |  |
| Parental death |  |  |  | <0.001 |
| No | 7670 (99.6) | 7330 (99.7) | 340 (97.4) |  |
| Yes | 34 (0.4) | 25 (0.3) | 9 (2.6) |  |

Values are expressed as n (%).

Supplementary Tables 2. Multivariate analysis of separation factors and IBS.

|  | Model 1 | | Model 2 | | Model 3 | |
| --- | --- | --- | --- | --- | --- | --- |
|  | OR (95% CI) | P value | OR (95% CI) | P value | OR (95% CI) | P value |
| Parental death |  |  |  |  |  |  |
| No | 1.0 |  | 1.0 |  | 1.0 |  |
| Yes | 1.7 (1.4, 2.0) | <0.0001 | 1.7 (1.4, 2.1) | <0.0001 | 1.6 (1.3, 2.0) | <0.0001 |

Model 1: unadjusted; Model 2: adjusted for age and sex; Model 3: adjusted for age, sex, BMI, school type, region, history of breastfeeding, long school accommodation, and prolonged school meals.
